# Development of a Bolometer Detector System for the NIST High Accuracy Infrared Spectrophotometer

**DOI:** 10.6028/jres.103.039

**Published:** 1998-12-01

**Authors:** Y. Zong, R. U. Datla

**Affiliations:** National Institute of Standards and Technology, Gaithersburg, MD 20899-0001

**Keywords:** cryogenic bolometer, infrared detector, linearity, noise, spatial response, spectrophotometer

## Abstract

A bolometer detector system was developed for the high accuracy infrared spectrophotometer at the National Institute of Standards and Technology to provide maximum sensitivity, spatial uniformity, and linearity of response covering the entire infrared spectral range. The spatial response variation was measured to be within 0.1 %. The linearity of the detector output was measured over three decades of input power. After applying a simple correction procedure, the detector output was found to deviate less than 0.2 % from linear behavior over this range. The noise equivalent power (NEP) of the bolometer system was 6 × 10^−12^ $W/\sqrt{\mathrm{Hz}}$ at the frequency of 80 Hz. The detector output 3 dB roll-off frequency was 200 Hz. The detector output was stable to within ± 0.05 % over a 15 min period. These results demonstrate that the bolometer detector system will serve as an excellent detector for the high accuracy infrared spectrophotometer.

## 1. Introduction

**Fig. 1:**
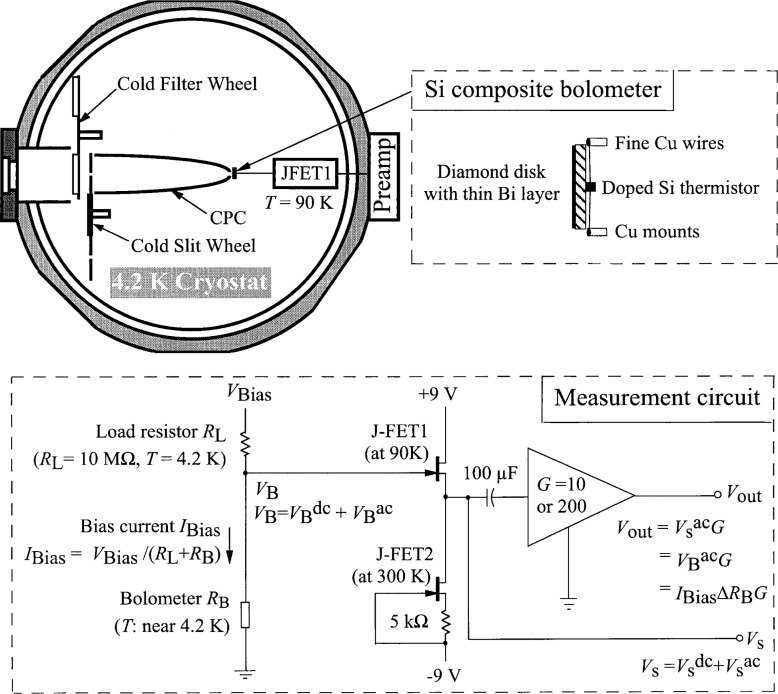
Broadband bolometer detector system.

A high accuracy, broad wavelength range (2 μm to 25 μm) dispersive infrared spectrophotometer is being developed at the National Institute of Standards and Technology [1]. The spectrophotometer is a single beam system whereby the reflectance or transmittance of a sample is measured as the ratio of the optical power detected with the sample in to that with the sample out. This is done for each wavelength setting. To cover the broad spectral range, the infrared detector for the spectrophotometer is a cryogenic bolometer detector system (Fig. 1) (Infrared Laboratories, Inc., 1808 E. 17th St., Tucson, AZ 85719, USA)^1^. It consists of a silicon composite bolometer, a compound parabolic concentrator (CPC) [2], a cold aperture, a cold filter, an infrared transmitting window, and a preamplifier. The silicon composite bolometer is a small (3 mm diameter) absorber thermally coupled to a heavily doped silicon thermistor. The gold-coated CPC, as shown in Fig. 1, has a 19 mm diameter front aperture, and was designed to concentrate the *f*/5 beam from the spectrophotometer onto the bolometer active area. The incorporation of the CPC has the advantage of increasing the acceptance aperture for the spectrophotometer beam, but it has the disadvantage of undesirable spatial and angular response. The cold aperture limits the field of view of the CPC-bolometer combination to view the monochromator exit slit only and to minimize any excess room-temperature radiation. The temperature of the cold aperture is 5 K during normal operation. Also, Fig. 1 shows the cold bandpass filter, mounted on a cold filter wheel, which limits the out-of-band radiation that reaches the detector. The temperature of the cold filter wheel is also 5 K during normal operation, and the infrared transmitting window, either KRS-5 or zinc selenide, preserves the vacuum integrity of the cryostat while transmitting infrared and visible radiation to the bolometer. Visible transparency of the window simplifies alignment and makes it possible to use visible light sources to test the bolometer detector system.

To perform high accuracy measurements with the infrared spectrophotometer, its bolometer detector system should have the following features: (1) The response of the bolometer detector system should have minimal spatial and angular dependence as the position of the beam on the detector shifts between the sample-in and sample-out cases. The wavelength dependence on responsivity is not critical as it cancels in the ratio of power detected between sample-in and sample-out positions at each wavelength setting. (2) The bolometer detector system response should be linear, with sufficient sensitivity and stability. Absolute responsivity is not needed because the spectrophotometer measures the ratio of the sample-in response to the sample-out response. Section 2 of this paper discusses the dependence of the response on the position and the angle of the incident beam. Section 3 discusses the nonlinearity of the detector system response and a technique for correcting nonlinearity of the detector system response. Section 4 describes the stability and noise of the detector system.

## 2. The Spatial and Angular Response

The response of the bolometer detector system has been shown to depend on the position and the angle of the incident beam [3]. Shifts in the position and the angle of the incident beam are possible as the sample is placed alternately into and out of the beam. Therefore, a variation in the spatial and angular response of the bolometer system could result in a systematic measurement error since the slit aperture of the bolometer detector system is under-filled by the exit slit of the monochromator.

The infrared spectrophotometer allows the operator to choose one of two measurement beam geometries at the sample: collimated beam or converging beam. The beam displacement and the incident angle at the detector will be different as the sample is moved into and out of the beam for measurements in these two cases.

The maximum beam displacement and angular shift can be estimated from the beam geometry. The estimated deviations for the two different beam geometries are given by

$$\begin{array}{l} \Delta d=(n-1)\theta f,\\ \Delta \alpha =(n-1)\theta \end{array}\}\;\text{collimated measurement beam},$$ (1)

and

$$\begin{array}{l} \Delta d=t\phi (n-1)/n,\\ \Delta \alpha =(n-1)\theta \beta \end{array}\}\;\text{converging measurement beam},$$ (2)

where Δ*d* is the displacement of the incident beam at the detector aperture, Δ*α* is the angular shift of the incident beam at the detector aperture, *n* is the refractive index of the sample, *t* is the thickness of a sample, *θ* is the wedge angle of the sample, *ϕ* is the tilt angle of the sample in order to avoid multi-reflection on optical elements (*ϕ* = 3.5 mrad for this spectrophotometer), *f* is the focal length of the collection optics, and *β* is the angular magnification of the collection optics (*β* = 3 for this spectrophotometer).

For example, in an extreme situation of measuring a wedged sample of germanium,

$$\begin{array}{l} n=4,\\ \theta =0.3\;\text{mrad},\\ t=5\;\mathrm{mm},\\ \phi =3.5\;\text{mrad}, \end{array}$$

and

$$\begin{array}{l} \Delta d\approx 0.18\;\mathrm{mm},\\ \Delta \alpha \approx 0.9\;\text{mrad} \end{array}\}\;\text{collimated measurement beam},$$ (3)

or

$$\begin{array}{l} \Delta d\approx 0.013\;\mathrm{mm},\\ \Delta \alpha \approx 2.7\;\text{mrad} \end{array}\}\;\text{converging measurement beam}.$$ (4)

Therefore, the largest expected displacement Δ*d* is 0.18 mm for measurements with the collimated beam and the largest incident angle shift Δ*α* is 2.7 mrad for measurements with the converging beam.

**Fig. 2:**
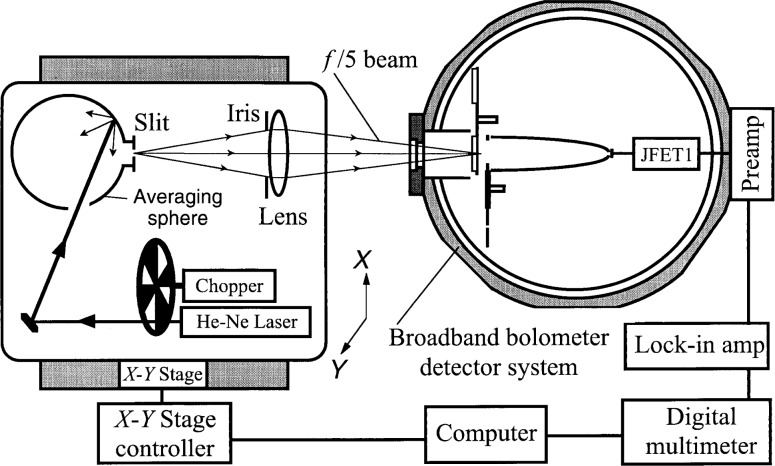
Experimental setup for the measurement of spatial response.

**Fig. 3:**
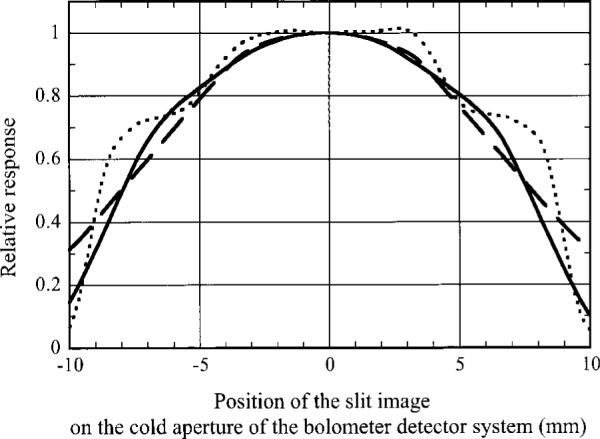
Spatial response of the bolometer detector system. Beam speed: *f*/5. Dotted curve: 1 mm × 1 mm slit image; Solid curve: 4.5 mm × 12 mm slit image scanned along the width of the slit image; Dashed curves: 4.5 mm × 12 mm slit image scanned along the height of the slit image.

The experimental setup used to test the spatial response of the detector system is shown in Fig. 2. All the components forming the optical beam were mounted on an *X*-*Y* translation stage. An integrating sphere irradiated with a laser was used to provide a uniform-brightness, unpolarized, slit source at its exit port. The slit was focused onto the cold aperture plane of the bolometer system. For these experiments the cold aperture wheel was used to select an 18 mm circular aperture in front of the CPC. The spatial response of the bolometer system was measured by scanning the beam using the *X*-*Y* translation stage; the results are shown in Fig. 3. The size of the image of the slit scanned across the cold aperture was chosen to be either 1 mm × 1 mm (dotted curve) or 4.5 mm × 12 mm (solid and dashed curves). The latter size slit image was chosen as a worst case since this is the largest slit size available in the spectrophotometer. The results in Fig. 3 demonstrate that when the beam is aligned to the center of the CPC, the output of the bolometer is not particularly sensitive to the displacement since the slope of the relative spatial response curve is near zero at the top. In the worst case the change of the response was less than 0.1 % for a beam displacement of Δ*d* = 0.18 mm from the center of CPC and did not exceed 1 % for displacements as large as 1 mm.

The angular response was checked by replacing the bolometer with a silicon photodiode in an equivalent geometry behind the CPC. The IR spectrophotometer beam geometry of the bolometer detector system was simulated by using a visible beam with *f*/5 geometry from a tungsten lamp with a lens and a 625 nm cut-on filter arrangement. This arrangement avoids the necessity to rotate the cryostat, which is more difficult to do if the bolometer itself is to be used for the measurements with the spectrophotometer as the source at IR wavelengths. A cut-on wavelength of approximately 625 nm was selected to reduce any strong angular dependence for reflectance from the gold CPC surface at wavelengths near its absorption edge. The change of the detector output was 0.15 % with a beam size of 1 mm diameter and a scan of the photodiode-CPC assembly through ± 0.9 mrad. For the extreme changes of the incidence angle Δ*α* = ± 2.7 mrad in the vertical plane or in the horizontal plane, the changes of the response were less than 0.86 % or 0.4 %, respectively, when the beam size was 3 mm (horizontal) × 12 mm (vertical). The 0.86 % change in the vertical plane due to the extreme change of incidence angle Δ*α* = ± 2.7 mrad is much larger than that would result from the maximum displacement of the slit image at the detector aperture. Therefore, in making converging beam measurements with the spectrophotometer, special care should be taken to orient wedged samples such that the wedge angle is in the horizontal plane, i.e., plane orthogonal to the slit height so that the sensitivity to beam angle is minimized. Also, because of the spatial and angular dependence of detector response, no displacement or angle change of the incident beam should be allowed when adjusting the incident power to measure the non-linearity of this system.

## 3. The Nonlinearity of the Bolometer System and Its Correction

The response of the bolometer system was measured and found to be nonlinear in earlier experiments [4, 5]. The main cause is the nonlinear dependence of bolometer resistance with temperature. In addition, the heat capacity and thermal conductivity of the bolometer components are generally also functions of temperature.

### 3.1 The Nonlinearity

The bolometer, shown schematically in Fig. 1, consists of a small heavily doped silicon thermistor mounted behind a 3 mm diameter chemical vapor depo sition (CVD) diamond disk which is used for absorbing incident radiation. Two fine copper wires connect the Si thermistor and serve as the thermal link to the cold plate at the base of the liquid helium cryostat. The front surface of the diamond disk is coated with a thin layer of bismuth to increase the absorption of radiation on the bolometer. Radiation incident on the bolometer increases the bolometer temperature causing a change in the resistance *R*_B_ of the Si thermistor. As illustrated in Fig. 1, the measurement circuit introduces a dc bias current *I*_Bias_ on the bolometer. The incident radiation is chopped and thus the signal is measured as an ac voltage across the bolometer. The bias causes some heating of the bolometer, and the bias voltage *V*_Bias_ and the load resistor *R*_L_ are used to set a proper operating point for the bolometer. The J-FET1 amplifier is operated at 90 K, and acts to match the impedance between the bolometer and external preamplifier at room temperature.

The phenomenological equation of the bolometer system is as follows:

$$V_{\text{out}}=GrP,$$ (5)

where *V*_out_ is the output voltage of the bolometer system, *G* is the gain of the preamplifier, *r* is the responsivity of the bolometer system, and *P* is the chopped incident power.

From Fig. 1, the bolometer output voltage *V*_B_ is

$$V_{B}=I_{\text{Bias}}\;R_{B}=\frac{V_{\text{Bias}}}{R_{L}+R_{B}}R_{B},$$ (6)

where *R*_B_ and *R*_L_ are the resistances of the bolometer and the load resistor, respectively. In this system *R*_L_ is 10 MΩ, and *R*_B_ is measured to be ≈ 10 MΩ with the background radiation power from outside of the cryostat cut off by blocking the window with an aluminum plate and placing a cold visible bandpass filter in front of the CPC.

The output voltage of the bolometer system can also be expressed as

$$V_{\text{out}}=GV_{B}^{\mathrm{ac}},$$ (7)

where *V*_B_^ac^ is the ac component of the bolometer output voltage *V*_B_.

**Fig. 4:**
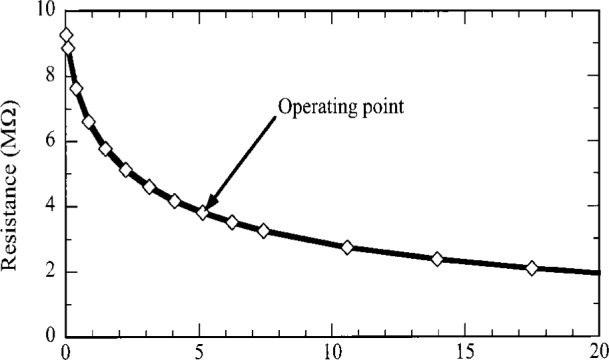
Nonlinear dependence of the resistance of the thermistor on the absorbed power.

From Eqs. (5), (6), and (7), the responsivity *r* will not be constant because of the following two factors. The first factor is the nonlinear dependence of the resistance *R*_B_ of the thermistor on the absorbed power [6], as shown in Fig. 4. Both the resistance and the slope of the curve decrease with increasing power. The responsivity of the bolometer detector system is proportional to the slope of the curve at the operating point. As the chopped input radiation power increases, the absorbed power of the bolometer also increases which decreases the responsivity of the thermistor. The second factor is the change of the bias current *I*_Bias_ of the bolometer resulting from the change of the resistance of the thermistor. This change of the bias current *I*_Bias_ tends to compensate the change of responsivity resulting from the first factor. The decrease of the resistance of the thermistor increases the bias current *I*_Bias_ in the thermistor, which in turn tends to increase the signal. This is also true of the ac component of the induced change in the bias current. This effect, sometimes referred to as electrothermal feedback, can also affect the frequency response of the bolometer [7, 8].

### 3.2 The Correction

**Fig. 5:**
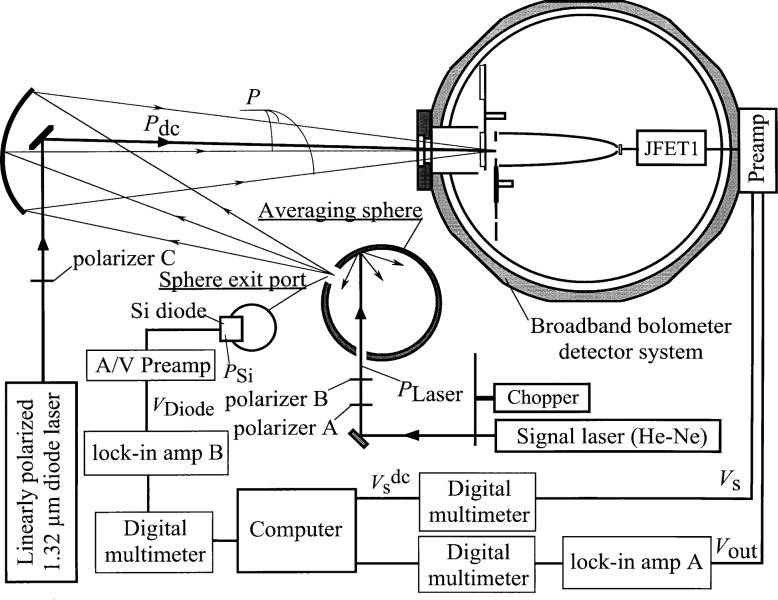
Experimental setup for the measurement of linearity and frequency response.

There are several traditional methods to measure the nonlinearity of a detector system. Two examples are measuring the transmittance of a sample at different input signal power levels [4], and adding two beams. J. P. Makai et al. tested the nonlinearity of this bolometer detector system earlier and suggested that the first factor mentioned above needed to be empirically studied and corrected [9]. They also suggested a scheme for insuring bias current stability in order to control the second factor. However, in later experiments we found that the changes in bias current could be used as an advantage as it somewhat compensates for the changes in the bolometer resistance. By using the experimental setup shown in Fig. 5 and some simple procedures, the nonlinearity of the bolometer detector system has been successfully corrected. The principle is to measure the responsivity at different incident power levels and different time average values of the bolometer output *V*_B_^dc^ and characterize the nonlinearity using these empirical data. We measure the time averaged voltage output *V*_s_^dc^ instead of *V*_B_^dc^ since (1) *V*_B_ is difficult to measure directly, and (2) the output of the J-FET1 amplifier *V*_s_ follows the output of the bolometer *V*_B_ exactly except for a small offset of ≈ 0.6 V due to the gate-source voltage of the J-FET1. The time averaging for *V*_s_^dc^ is accomplished over a time period of approximately 3 s by sampling 200 times and averaging these data.

In the experimental setup shown in Fig. 5, a chopped, high power (25 mW) He-Ne signal laser beam is used in order to measure the nonlinearity in a large dynamic range. The gain of the preamplifier of the bolometer system was set to 10 accordingly. Polarizer A is used as a crossed polarizer to change the input signal power *P* and polarizer B is used to select the polarization direction and keep it unchanged as the incident power is changed by rotating polarizer A. An unchanged polarization direction is necessary to avoid any signal change resulting from the change of the polarization direction. The averaging sphere is used to keep the position and angle of the beam focused into the bolometer system unchanged during the measurement process.

A Si photodiode was placed at the exit aperture of the sphere to monitor a fraction of the power of the signal laser beam transmitted through the chopper and the two polarizers A and B. This laser beam is directed close to the exit port of the averaging sphere to avoid the first reflection reaching the photodiode. The gain of the transimpedance current preamplifier of the photodiode was set at 200 V/μA and was unchanged in the process of the measurement.

A linearly polarized 1.32 mm diode laser and polarizer C (Fig. 5) were used to vary the dc background radiation incident on the bolometer in order to measure the output *V*_out_ at different values of *V*_s_^dc^ for each input power setting. Two identical lock-in amplifiers A and B were used to measure the output *V*_out_ of the bolometer system and the output of the Si photodiode, respectively. The lock-in amplifiers had been characterized for their linearity and offset.

**Fig. 6:**
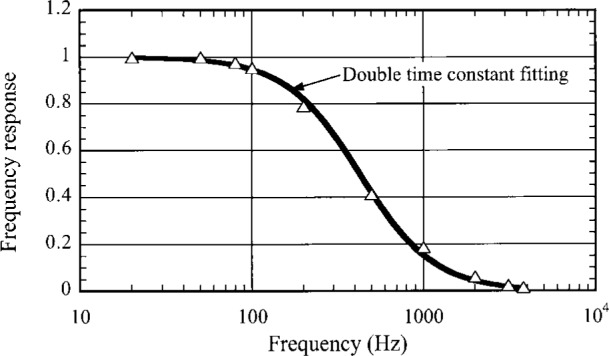
Frequency response of the bolometer detector system.

The method used to correct the nonlinearity is based on the following two assumptions. The first assumption is that the bolometer has no photoconductive response from the signal He-Ne laser beam. Since much of the He-Ne laser light, which is not absorbed by the bolometer absorber, irradiates the silicon thermistor (see Fig. 1), it is possible that the bolometer would exhibit some photoconductive effect. In the setup shown in Fig. 5, this was checked by measuring the frequency response of the bolometer system; the results are shown in Fig. 6. As can be seen from the figure, the high frequency signal approaches zero, and the signal at 3.8 kHz is only 1.6 % of that at 80 Hz. Therefore, we can conclude that the response of the bolometer is dominated by the thermal effect because the bolometer detector is unable to respond to variations of a few kilohertz in the input power. This is as expected based on its known thermal response. If the response had been due to a photoconductive effect, the signal would not have approached zero because we expect the photoresponse to be much faster than the thermal effect. The second assumption is that the nonlinearity of the Si photodiode is negligible compared to the nonlinearity of the bolometer system. This is a safe assumption because the nonlinearity of the Hamamatsu S1227-1010 Si photodiode is less than 0.1 % over the power ranges encountered [10, 11].

To obtain the nonlinearity correction function, we let *r*_Si_ be the responsivity of the photodiode, *G*_Si_ be the gain of its preamplifier, and *P*_Si_ be the chopped input signal power to the photodiode. The output voltage *V*_Diode_ of the silicon photodiode’s transimpedance amplifier is given by

$$V_{\text{Diode}}=G_{\mathrm{Si}}r_{\mathrm{Si}}P_{\mathrm{Si}}.$$ (8)

**Fig. 7:**
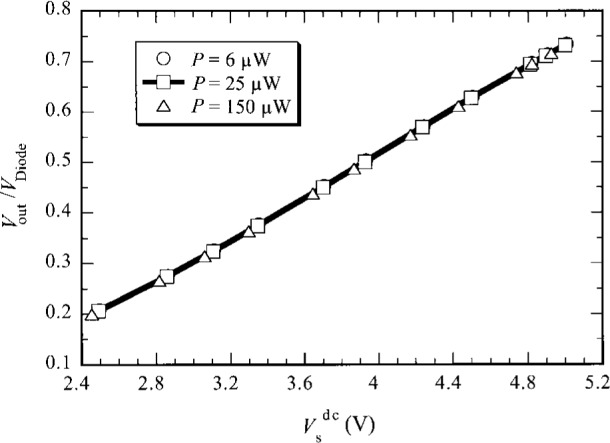
The ratio of bolometer to photodiode signals versus *V*_s_^dc^ for various applied signal power levels.

The ratio of the bolometer output to the photodiode signal is

$$\frac{V_{\text{out}}}{V_{\text{Diode}}}=\frac{GrP}{G_{\mathrm{Si}}r_{\mathrm{Si}}P_{\mathrm{Si}}}=\frac{Gr\alpha_{B}}{G_{\mathrm{Si}}r_{\mathrm{Si}}\alpha_{\mathrm{Si}}},$$ (9)

where *P* = *α*_B_ *P*_Laser_, *P*_Si_ = *α*_Si_ *P*_Laser_, and *P*_Laser_ is the chopped input signal power of the laser beam into the sphere, which need not be very stable in time because *V*_out_ and *V*_Diode_ are measured simultaneously. The geometry factors *α*_B_ and *α*_Si_ represent the fractions of laser radiation power impinging on the bolometer and the photodiode, respectively. In this experiment *r*_Si_, *G*_Si_, *G*, *α*_B_, and *α*_Si_ are kept constant. The responsivity *r* is obtained by measuring *V*_out_/*V*_Diode_ at different *V*_s_^dc^ and *P* levels. However, the dependence of the responsivity *r* on *P* is found to be negligible. This can be seen in Fig. 7, which shows the ratio of *V*_out_/*V*_Diode_ as a function of *V*_s_^dc^ at different signal power levels *P*: 6 μW, 25 μW and 150 μW. It shows that all the measured data at three different signal power levels are almost on the same line. This demonstrates that the contribution of varying *P* to the change of the ratio of *V*_out_/*V*_Diode_ is negligible compared to that of *V*_s_^dc^. Therefore the responsivity *r* is a function of *V*_s_^dc^ only.

Let the responsivity *r* normalized to its value at *V*_s_^dc^ = 5 V be the nonlinearity correction function *R*. We then have

$$R=\frac{r}{r|V_{s}^{\mathrm{dc}}=5\;V}.$$ (10)

**Fig. 8:**
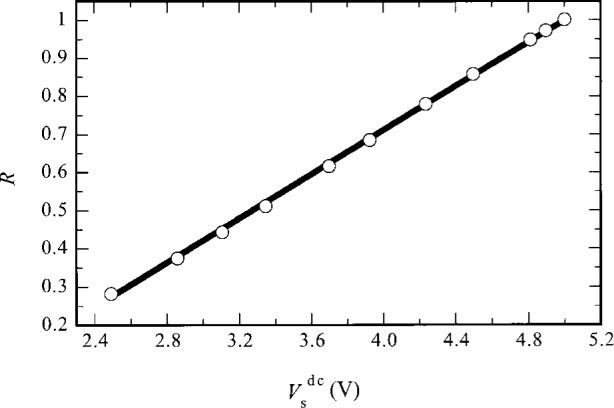
The nonlinearity correction function *R*. *R*(solid line) = – 0.4573 + (0.292 V^−1^) *V*_s_^dc^.

Figure 8 shows the regression function for *R*, which is obtained by fitting a set of values of *R* at different *V*_s_^dc^ levels. Each datum in Fig. 8 is an average of 200 data readings over a time period of 3 s. The standard deviation for each data point is much smaller than the radius of the symbol used in the graph. The value of *R* and its uncertainty (two standard deviation estimate) Δ*R* at any *V*_s_^dc^ level can be obtained from the functional relationships [12]

$$R=-0.4573+(0.292V^{-1})V_{s}^{\mathrm{dc}},$$ (11)

and

$$\Delta R=2\sqrt{1.348\times 10^{-5}-(5.52\times 10^{-6}V^{-1})V_{s}^{\mathrm{dc}}+(5.7\times 10^{-7}V^{-2}){\left(V_{s}^{\mathrm{dc}}\right)}^{2}}.$$ (12)

The uncertainty Δ*R* in Eq. (12) includes only the component associated with the fitting and the scatter in the data. Another component arising from long-term stability of the bolometer detector system over a period of years is still to be determined. Yet another component is that associated with the drift of *V*_s_^dc^. This is because the above method for the nonlinearity correction is based on the assumption that the value of *V*_s_^dc^ depends only on the absorbed radiant power at the bolometer chip. However, a small drift of *V*_s_^dc^ is possible because of the drift of bias voltage *V*_Bias_ of the bolometer, and the drift of the gate source voltage *V*_GS_ of the J-FET1. The drift of *V*_Bias_ is less than 0.01 % because of the highly stable and low noise constant voltage power supply (+ 15 V) employed in the preamplifier circuit. While the drift of *V*_s_^dc^ (when *V*_s_^dc^ = 5 V) was observed to be less than 0.05 V based on the data of several tests performed through a one-year period, the correction error of the responsivity *r* resulting from this drift of *V*_s_^dc^ is 1.44 % according to Eq. (10).

By substituting Eq. (10) in Eq. (5), we have the bolometer output characterized for its nonlinearity in response to incident power *P*:

$$V_{\text{out}}=GR(r|V_{s}^{\mathrm{dc}}=5\;V)P.$$ (13)

In Eq. (13)*G* and *r*|*V*_s_^dc^ = 5 V are constants whose values are known. The nonlinearity correction function *R* can be obtained by measuring *V_s_*^dc^ and using Eq. (11). Thus, the incident power *P* can be obtained from the measured output *V*_out_. The uncertainty in obtaining *P* depends on the uncertainty of the nonlinearity correction function *R*.

**Fig. 9:**
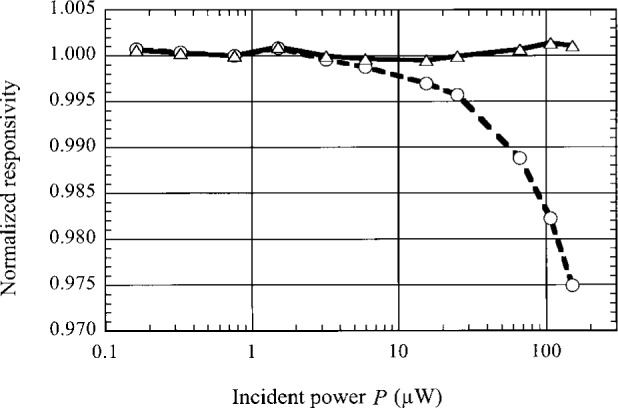
Verification of the effectiveness of the nonlinearity correction function *R*. The curve marked with symbol “o” and the curve marked with symbol “Δ” are the plots of the data of responsivity *r* and responsivity *r*|*V*_s_^dc^ = 5 V normalized to their respective values at *P* = 0.76 μW.

To verify the effectiveness of the nonlinearity correction function *R*, a set of data for the bolometer system output *V*_out_ and incident power *P* were obtained over three decades of incident power at *V*_s_^dc^ ≈ 5 V. The responsivity *r* and the responsivity at *V*_s_^dc^ = 5 V, i.e., *r|V*_s_^dc^ = 5 V, were calculated for each power level *P* using Eq. (5) and Eq. (13), respectively. The responsivity data normalized to their respective values at *P* = 0.76 W are plotted in Fig. 9. The variation of *r* is about 2.5 %, while the variation of *r*|*V*_s_^dc^ = 5 V is only 0.2 % for three decades of the incident power *P*. This demonstrates that *R* is very effective as the nonlinearity correction, and the nonlinearity over three decades of incident power *P* is reduced from 2.5 % to 0.2 %. In this case the relative nonlinearity correction uncertainty Δ*R*/*R* is only 0.07 % according to Eqs. (11) and (12), which is much smaller than the above nonlinearity value 0.2 %. This is due to the non-linearity of the lock-in amplifier used in the measurements.

### 3.3 Application

As the spectrophotometer measures the ratio of detected power between the sample-in case to the sample-out case, the measurement equation of the ratio is obtained from Eq. (13):

$$\frac{P(\text{sample}-\text{in})}{P(\text{sample}-\text{out})}=\frac{V_{\text{Out}}(\text{sample}-\text{in})}{V_{\text{Out}}(\text{sample}-\text{out})}\frac{R(\text{sample}-\text{out})}{R(\text{sample}-\text{in})}.$$ (14)

By measuring the *V*_s_^dc^ and *V*_out_ for the sample-in and sample-out cases, the ratio of detected power between the two cases can be obtained. The absolute value of the responsivity *r* is not needed. Only the relative value, i.e., the value of the nonlinearity correction function *R*, is necessary when using this bolometer detector system for the spectrophotometric measurements.

## 4. Stability and Noise Equivalent Power (NEP) of the Bolometer

**Fig. 10:**
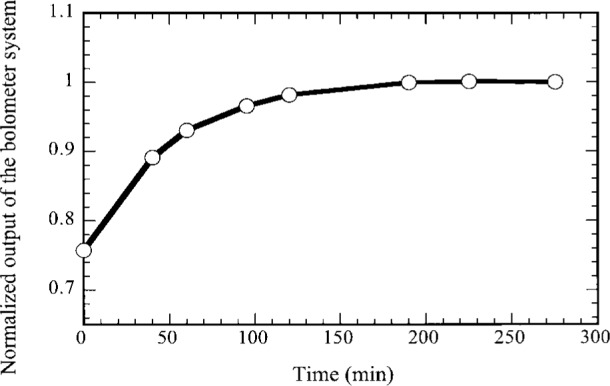
Stability of the bolometer detector system. “0” was the time when liquid helium was transferred into the cryostat and the preamplifier was turned on.

The stability of the bolometer detector system was measured with the experimental setup used for the non-linearity measurement (Fig. 5). Figure 10 shows that the output of the bolometer detector system changes rapidly in the first 3 h. This is probably due to the temperature drift of the J-FET1 in the preamplifier (see Fig. 1). The J-FET1 is mounted on a heat-resistant column with a very small electric heater. After 3 h the temperature of the J-FET1, as well as the output, was stable. The stability of the bolometer system was measured for 15 min after such a stabilization, and the output drift was only 0.05 %.

The NEP of the bolometer system was found to be 6 × 10^−12^ $W/\sqrt{\mathrm{Hz}}$ based on the measurements of the noise density and the sensitivity. The absolute sensitivity of the bolometer system was measured by using a 1.32 μm wavelength diode laser calibrated by a power meter. The diameter of the laser beam was adjusted to approximately 10 mm diameter to decrease the effect of the position dependence on the input beam. The chopping frequency was set to 80 Hz, and the gain of the preamplifier was set to 200. Under these conditions, the sensitivity of the bolometer system was 1 × 10^6^ V/W. The noise density of the bolometer system was measured by using a spectrum analyzer.

## 5. Conclusion

The bolometer detector system developed for the high accuracy infrared spectrophotometer at NIST was discussed. The spatial dependence on the incident *f*/5 light beam was less than 0.1 % within the displacement of 0.18 mm. Major factors causing the nonlinearity of the bolometer detector system response were corrected. The nonlinearity at *V*_s_^dc^ ≈ 5 V was reduced from 2.5 % to 0.2 % over three decades of input power after the correction is applied. The stability over 15 min was better than 0.05 %. In general, it was found that the bolometer detector system can serve the needs of the infrared (2 μm to 25 μm) spectrophotometer used at NIST.

## Footnotes

1 Certain commercial equipment, instruments or materials are identified in this paper to foster understanding. Such identification does not imply recommendation or endorsement by the National Institute of Standards and Technology, nor does it imply that the materials or equipment identified are necessarily the best available for the purpose.
